# Quercetin relieves compression-induced cell death and lumbar disc degeneration by stabilizing HIF1A protein

**DOI:** 10.1016/j.heliyon.2024.e37349

**Published:** 2024-09-02

**Authors:** Junxiao Ren, Rui Xin, Xiaoping Cui, Yongqing Xu, Chuan Li

**Affiliations:** a The First Clinical Medical College of Yunnan University of Chinese Medicine, Kunming, 650500, Yunnan, China; b Chongqing Fengdu County Traditional Chinese Medicine Hospital, Chongqing, 408200, China; c The 920th Hospital of Joint Logistics SupportForce of PLA, Kunming, 650032, Yunnan, China; d Engineering Laboratory of Peptides of Chinese Academy of Sciences, Kunming, Yunnan, 650201, China

**Keywords:** Shaoyao-Gancao decoction (SGD), Lumbar disc degeneration (LDD), HIF1A, Quercetin, nucleus pulposus mesenchymal stem cells (NPMSCs)

## Abstract

**Background:**

Lumbar disc degeneration (LDD) is a prevalent condition characterized by the decreased viability and functional impairment of nucleus pulposus mesenchymal stem cells (NPMSCs). Shaoyao-Gancao decoction (SGD), a traditional Chinese medicine formula, has been used to treat LDD, but its active components and mechanisms are unclear.

**Methods:**

An integrative network pharmacology and transcriptome analysis were conducted to identify bioactive compounds in SGD that could target LDD. NPMSCs were cultured under mechanical compression as a cellular model of LDD. A rat model of annulus fibrosus needle-puncture was established to induce intervertebral disc degeneration. The effects of quercetin, a predicted active component, on alleviating compression-induced NPMSC death and LDD were evaluated *in vitro* and *in vivo*.

**Results:**

The analysis identified hypoxia-inducible factor 1-alpha (HIF1A) as a potential target of quercetin in LDD. HIF1A was upregulated in degenerated human disc samples and compression-treated NPMSCs. Quercetin treatment alleviated compression-induced oxidative stress, apoptosis, and loss of viability in NPMSCs by stabilizing HIF1A. The protective effects of quercetin were abrogated by HIF1A inhibition. In the rat model, quercetin ameliorated intervertebral disc degeneration.

**Conclusion:**

Our study identified HIF1A as a protective factor against compression-induced cell death in NPMSCs. Quercetin, a bioactive compound found in the traditional Chinese medicine formula SGD, improved the survival of NPMSCs and alleviated LDD progression by stabilizing HIF1A. Targeting the HIF1A pathway through natural compounds like quercetin could represent a promising strategy for the clinical management of LDD and potentially other degenerative disc diseases.

## 1 Introduction

Lumbar disc degeneration (LDD) is a prevalent pathological condition worldwide, significantly impacting the quality of life for patients and imposing a substantial financial burden on society [1,2]. Various factors could contribute to the development of LDD, including genetic predisposition [3], degradation of collagen [4], excessive mechanical strain [5], and compromised proliferation of nucleus pulposus cells (NPCs) [6]. Nucleus pulposus mesenchymal stem cells (NPMSCs), also referred to as progenitor cells of the nucleus pulposus (NP), possess similar differentiation capabilities comparable to mesenchymal stem cells (MSCs). During LDD, there is a notable decline in the viability, quantity, and functional potency of NPMSCs [7,8]. Due to their capacity for multidirectional differentiation and tissue specificity [9,10], NPMSCs exhibit potential superiority over non-intervertebral disc MSCs in terms of NPC differentiation and emerge as a prospective therapeutic cell source for LDD [11]. Investigating the impact of adverse microenvironment conditions on NPMSCs, including compression, may open up possibilities for intervening in and restoring damaged NP tissues. This approach holds great promise in the treatment of LDD.

Shaoyao-Gancao decoction (SGD) is a traditional Chinese medicinal formula that contains two therapeutic components: white peony (*Paeonia lactiflora*) and licorice (*Glycyrrhiza uralensis*) [12]. This formula has been demonstrated to maintain functional homeostasis in the liver and spleen while offering pain-relieving effect [13,14]. In clinical settings, it serves as a medical intervention for managing diverse forms of visceral pain and spasms in the gastrocnemius muscle attributed to blood insufficiency [12,15]. The analgesic effects of SGD may be attributed to the inhibition on the production of oxygen free radicals (OFR), as well as the anti-inflammatory activity [16,17]. SGD also exhibits an antispasmodic activity, wherein its mechanism primarily involves the hindrance of calcium ion channels within the smooth muscle, thereby diminishing the force of smooth muscle contractions [18]. Apart from that, a growing body of evidence highlights the neuroprotective effect of SGD, mainly through the antioxidant and anti-apoptotic effects [19,20]. Nevertheless, its therapeutic potential on LDD has not yet been explored.

There are abundant species of bioactive compounds present in SGD, such as paeoniflorin, glycyrrhetinic acid, albiflorin, liquiritin and liquiritigenin [21]. It is intuitive to speculate that diverse components present in SGD could target different protein targets and signaling pathways. For instance, SGD suppresses the inflammation in polycystic ovary syndrome by inhibiting Toll-like receptor 4/nuclear factor kappa B (TLR4/NF-κB) Pathway [17]. In a mouse model of Alzheimer's disease, SGD exerted a neuroprotective function by reducing NOD-like receptor protein (NLRP)-1 and NLRP-3 [16]. Nonetheless, the major components accounting for the therapeutic activity of SGD remain elusive. Unraveling the responsible constituents in SGD for various pathophysiological conditions can offer valuable insights into the mechanism of action and protein target.

Given the widely reported therapeutic activities of SGD, especially its antioxidant and anti-apoptotic effects [19,20], and the involvement of oxidative stress and intervertebral disc cell death in LDD [22,23], we hypothesized that SGD may possess a potential beneficial effect against LDD progression. To investigate the therapeutic potential of SGD against LDD, an integrated approach was employed. Network pharmacology and transcriptomics analyses were first conducted to identify potential bioactive compounds in SGD targeting LDD and their molecular targets, leading to the identification of quercetin as a compound stabilizing hypoxia-inducible factor 1-alpha (HIF1A) protein. The ability of quercetin to alleviate compression-induced oxidative stress and apoptosis was assessed in NPMSCs. The role of the HIF1A pathway in mediating quercetin's protective effects was investigated through pharmacological inhibition. Finally, the therapeutic efficacy of quercetin was assessed in a rat model of intervertebral disc degeneration induced by annulus fibrosus needle puncture. Our findings pinpoint quercetin, an active component of SGD, as a promising agent to ameliorate LDD progression by stabilizing HIF1A protein.

## 2 Materials and methods

### 2.1 Bioinformatics analysis

The screening of active ingredients in Chinese medicine formula Shaoyao-Gancao decoction (SGD) was conducted through TCMSP database, BATMAN-TCM database, and TCMID database, based on DL value ≥ 0.18 and OB value ≥ 30 %. Gene targets related to lumbar disc degeneration (LDD) were retrieved from Genecard database, OMIM database, and TTD database. Cytoscape 3.9.0 software was employed to visualize the interactions of disease targets and active ingredients. The protein-protein interaction (PPI) network was constructed using the protein pair information retrieved from the string database. The plugin cytohubba module of Cytoscape 3.9.0 software was used to score the target proteins. To determine the biological function of candidate targets, Gene Ontology and KEGG pathway enrichment analysis was performed using BioProfiler package in R4.1.3 software, and the significant enrichment results were based on the criteria of corrected P value greater than 0.05 and count value greater than 2. To profile the differentially expressed genes in LDD, the microarray datasets GSE56081 and GSE167199 related to LDD samples were retrieved GEO database. These data were subjected to differential expression analysis using the limma package of R4.1.3 software, using a threshold of the absolute value of Log2 (Fold Change) greater than 1 and the adjusted P value less than 0.05.

### 2.2 Molecular docking

The molecular docking process was conducted as follows: 1. Active ingredients were searched on PubChem's official website (https://PubChem.ncbi.nlm.nih.gov/) using their English chemical names. The two-dimensional structure of each active ingredient was downloaded and saved in SDF format. 2. The SDF file of each active ingredient was then uploaded to ChemBio3D Ultra 14.0 software to construct their respective three-dimensional structures. The resulting structures were saved as MOL2 format files. 3. Core target genes were searched on Uniprot's official website (https://www.UniProt.org/) using relevant keywords. The Protein ID of the core target genes was obtained. 4. The 3D structure PDB format of the core target protein was downloaded from the Protein Data Bank (PDB) using the Protein ID as the keyword. 5. Water and small molecule ligands were removed from the PDB file using Pymol software, and the modified file was saved as a PDB file. 6. The active pockets of the protein receptor were plotted and the active pocket parameters were adjusted using AutoDockTools-1.5.6 software. 7. Docking was performed using AutoDock Vina, with each object being docked 20 times. The optimal result was determined based on the lowest free energy. 8. The optimal model PDB file was loaded into Discovery Studio 2016client (permission required) to calculate the interaction forces between residues in the docking region. 9. The three-dimensional and two-dimensional figures of the forces acting on the docking region were then outputted. 10. The energy of the drug-gene interaction was measured in kcal/mol.

### 2.3 Isolation and culturing of NPMSCs

Human nucleus pulposus specimens were collected from six patients who underwent discectomy for degenerative disc disease. The usage of huamn samples gained the approval of the medical ethics committee of The 920th Hospital of Joint Logistics SupportForce of PLA (Lunshen 2024-005(Ke)-01). The NP tissues were isolated from patients with lumbar disc hernia using a dissecting microscope. They were then washed with phosphate-buffered saline (PBS) and dissociated for 4 h in 0.25 % type II collagenase (Sigma, St. Louis, MO, USA) at 37 °C. Following the digestion, the samples underwent two rounds of centrifugation at 300×*g* for 5 min before being suspended and cultured in a complete medium for mesenchymal stem cells (MSCs) (Cyagen, Guangzhou, China) at 37 °C in a humidified atmosphere with 5 % CO_2_. The culture medium was refreshed every 3 days. When reaching 80–90 % confluence, the cells were subcultured at a ratio of 1 : 3 [24].

To construct a mechanical compression stress model, a protocol involving the application of pressure in cell culture was established using a stainless-steel pressure vessel connecting to a compression apparatus [25]. The pressure vessel was specifically designed to endure pressures exceeding 1.5 MPa. NPMSCs were carefully positioned on cell culture plates and subjected to a precisely controlled pressure of 1.0 MPa for different periods. To preserve optimal humidity levels, a small quantity of distilled water was introduced into the pressure vessel, and the entire setup was then placed inside a 37 °C incubator. For comparison, control cells were also incubated at 37 °C under a 5 % CO_2_ atmosphere but at a significantly lower pressure of 0.1 MPa for a duration of 48 h.

### 2.4 RT-qPCR

The initial step involved the extraction of total RNA from approximately 1 x 10^6 cells using the TRIzol reagent (Sigma-Aldrich) according to the manufacturer's instructions. RNA quantity and purity were assessed using a NanoDrop spectrophotometer (Thermo Scientific). 1 μg of total RNA was then reverse transcribed into cDNA using the High-Capacity cDNA Reverse Transcription Kit (Merck, Darmstadt, Germany) following the kit protocol. Quantitative real-time PCR (qPCR) reaction was performed using the SYBR Green PCR master mix (KAPA Biosystems, Wilmington USA) on a QuantStudio 6 Flex Real-Time PCR System (Applied Biosystems). The 20 μL qPCR reaction mixture consisted of 10 μL SYBR Green master mix, 1 μL of 10 μM forward and reverse primers, 2 μL of cDNA template, and 7 μL nuclease-free water. The cycling conditions were as follows: an initial denaturation at 95 °C for 3 min, followed by 40 cycles of denaturation at 95 °C for 10 s, annealing at 60 °C for 30 s, and extension at 72 °C for 30 s. A melting curve analysis was performed at the end of each run to verify the specificity of the PCR products. Relative gene expression was determined using 2^(-delta delta CT) approach [26]. The following primers were used:

IL-6, F-CCTGAACCTTCCAAAGATGGC, R-TTCACCAGGCAAGTCTCCTCA;

HIF1A, F-GAACGTCGAAAAGAAAAGTCTCG, R-CCTTATCAAGATGCGAACTCACA;

Beta-actin, F-CATGTACGTTGCTATCCAGGC, R-CTCCTTAATGTCACGCACGAT.

### 2.5 Immunoblotting

For protein extraction, approximately 1 x 10^6 cells were lysed in radioimmunoprecipitation assay (RIPA) buffer (Beyotime, Beijing, China) containing 1 % phosphatase and protease inhibitor cocktails (Beyotime). The lysates were incubated on ice for 30 min and centrifuged at 12,000×*g* for 15 min at 4 °C. The supernatants were collected, and protein concentrations were determined using a bicinchoninic acid (BCA) protein assay kit (Sigma-Aldrich) according to the manufacturer's instructions. For Western blotting, 15 μg of protein samples were separated by 12 % SDS-polyacrylamide gel electrophoresis and then transferred onto polyvinylidene difluoride (PVDF) membranes (Millipore, CA, USA). The membranes were blocked with 5 % non-fat dry milk in Tris-buffered saline containing 0.1 % Tween-20 (TBST) for 1.5 h at room temperature. After blocking, the membranes were incubated overnight at 4 °C with primary antibodies against HIF1A (ab16066, 1:1000 dilution, Abcam, Cambridge, UK), IL-6 (ab9324, 1:1000 dilution, Abcam), or β-actin (loading control, 1:5000 dilution, Sigma-Aldrich) diluted in TBST containing 5 % non-fat milk. Following primary antibody incubation, the membranes were washed three times with TBST and then incubated with horseradish peroxidase (HRP)-conjugated secondary antibodies (1:5000 dilution, Cell Signaling Technology) for 1 h at room temperature. After washing, the protein bands were visualized using an Ultra High Sensitivity ECL Kit (MedChemExpress, Shanghai, China) and the ChemiDoc Imaging System (Bio-Rad). Densitometric analysis of the protein bands was performed using ImageJ software (NIH, MA, USA).

### 2.6 Cell viability assay

The cellular viability of NPMSCs was assessed after applying a compressive pressure of 1.0 MPa for different durations of 0, 12, 24, 36, or 48 h or after the treatment with quercetin (5, 10, 20 μM). The doses of quercetin were selected since the treatment of these consecrations alone did not induce toxicity in NPMSCs. The cell count kit-8 (CCK-8) assay (Dojindo, Tokyo, Japan) was employed for this purpose, following the instructions provided by the manufacturer. Specifically, cells were seeded in 96-well plates at a density of 6 × 10^3^ cells per well and allowed to attach overnight. The following day, cells were treated with the desired compounds or subjected to compression as per the experimental design. At the designated time points, 10 μL of CCK-8 solution was added to each well containing 100 μL of culture medium, and the plates were incubated for 2 h at 37 °C in a humidified CO_2_ incubator. After incubation, the plates were briefly agitated on an orbital shaker to ensure homogeneous distribution of the formazan dye produced by the reduction of CCK-8 by dehydrogenases in viable cells. The absorbance was measured at 450 nm using a microplate reader (BioTek Instruments, USA). The measured absorbance values were directly proportional to the number of viable cells in each well [27].

### 2.7 Flow cytometry analysis of apoptotic event and mitochondrial reactive oxygen species (ROS)

To detect apoptotic events, a total number of 5 × 10^5^ cells were suspended in 200 μL of annexin-V staining buffer containing 4 μL of propidium iodide (0.5 mg/mL, Sigma-Aldrich), and 2 μL of Annexin V-Fluorescein Isothiocyanate dye (Sigma-Aldrich). The cells were subjected to a 15-min staining at ambient temperature. For mitochondrial ROS analysis, cells were stained with 10 μM mitoSOX Red dye (ThermoFisher Scientific, CA, USA) for 30 min [28]. Cellular analysis was carried out employing the BD Accuri C6 flow cytometry instrument (BD Biosciences, CA, USA).

### 2.8 Protein stability assay

The cells under different experimental conditions were subjected to incubation with cycloheximide (CHX, HY-12320, MedChemExpress, Shanghai, China) at a concentration of 10 μg/ml for varying durations of 0, 2, 4, and 6 h. At the indicated time intervals, RIPA buffer was used to lyse the cells, following which immunoblotting was performed to assess the protein levels of HIF1A, with β-actin as the internal control. The relative protein levels at different time points were normalized against that of 0 h time point [29].

### 2.9 HIF1A transcription activity assay and ROS detection

After pre-treatment with mechanical compression and/or chemical compounds, the intracellular transcriptional activity of HIF-1α was assessed using the Human HIF-1 alpha Transcription Factor Activity Assay Kit (RayBiotech, CA, USA), according to the manufacturer's protocol. Briefly, nuclear protein extracts were prepared from treated cells using the nuclear extraction reagents provided in the kit. The protein concentration of the nuclear extracts was determined by the Bradford assay (Bio-Rad). For the transcription factor activity assay, 10 μg of nuclear extract was incubated with an immobilized HIF-1α consensus binding oligonucleotide in a 96-well plate. After incubation, the plate was washed to remove unbound proteins, and a primary antibody specific for HIF-1α was added and incubated further. Following a wash step, an HRP-conjugated secondary antibody was added and incubated. The plate was washed again, and a developing solution was added for colorimetric detection. The enzymatic reaction was stopped by adding a stop solution, and the absorbance was measured at 450 nm using a microplate reader (BioTek Instruments) [30].

The levels of intracellular ROS was measured utilizing a ROS detection kit (Sigma-Aldrich, MO, USA). To achieve this, NPMSCs were exposed to 2,7-dichlorofluorescin diacetate (DCFHDA) in a light-deprived environment at a temperature of 37 °C for a duration of 30 min [31]. The relative signals were recorded using a Synergy H1 spectrophotometer (Bio-Tek, CA, USA).

### 2.10 Electron microscope

NPMSCs were exposed to 2.5 % glutaraldehyde in PBS for 2 h at room temperature. After post-fixation with 1 % osmium tetroxide for an additional 2 h, the cells underwent dehydration steps in ethanol and were then infiltrated and embedded in Epon 812. Utilizing an ultramicrotome (EM UC7, Leica, Germany), ultra-thin sections were obtained and stained with uranyl acetate and lead citrate [32]. Finally, the sections were examined using a Tecnai G2 20TEM (FEI Company, USA).

### 2.11 Animal experiments

Sprague-Dawley rats (aged 3 months, 450–500g) were subjected to anesthesia via i.p. injection of Pentobarbital sodium (50 mg/kg) before the establishment of LDD model [33]. The caudal region of the rat was confirmed by palpating, and caudal vertebrae (Co7, Co8, and Co9) were located through experimental radiography. Next, the caudal vertebrae were cleaned with povidone iodine and secured onto the Ilizarov instrument. To fix them in place, percutaneous insertion of 0.7 mm diameter Kirschner needles was performed on the 7th and 9th caudal vertebrae, at a perpendicular angle to the caudal axis. These needles were then connected longitudinally using four threaded rods, which were fixed to the aluminum ring. Following that, four 0.50 N/mm calibration springs were attached to each bar, tightness adjusted from the distal side to create a predetermined compressive stress of approximately 1.3 MPa for 4 weeks. On week 4, the pressure from Ilizarov apparatus was relieved for another two weeks until tissue collection. In the sham group, no external mechanical load was applied by the installation of the compression equipment during the whole period of experiment. In the drug administration group, after 4 weeks of compression, the pressure was relieved and quercetin (20 mg/kg/day, [34,35]) was injected into the intervertebral disc of Co8/Co9 twice a week. After 2 weeks, the rats were euthanized by cervical dislocation and intervertebral disc Co7/8 and Co8/9 tissues were subjected to histological analysis using an Hematoxylin&eosin staining kit (Beytotime, Beijing China) and a Safranin O-fast Green (S-O) staining kit (Solarbio, Beijing China). The animal protocol was approved by the animal use ethics committee of The 920th Hospital of Joint Logistics SupportForce of PLA (Lunshen 2024-005(Ke)-01).

### 2.12 Statistics

The statistical analysis for this study was conducted using GraphPad Prism 6 software (GraphPad Software Inc, San Diego, CA, USA). All the data were acquired from a minimum of three individual experiments and depicted as the mean ± standard deviation (SD). To compare multiple groups of data, one-way or two-way analysis of variance (ANOVA) test was adopted together with Tukey's post hoc test. Meanwhile, Student's t-tests were employed for analyzing parameters associated with two groups. The statistical significance level was set at *P* < 0.05.

## 3 Results

### 3.1 Identification of potential protein targets of SGD formula

**Fig. 1:**
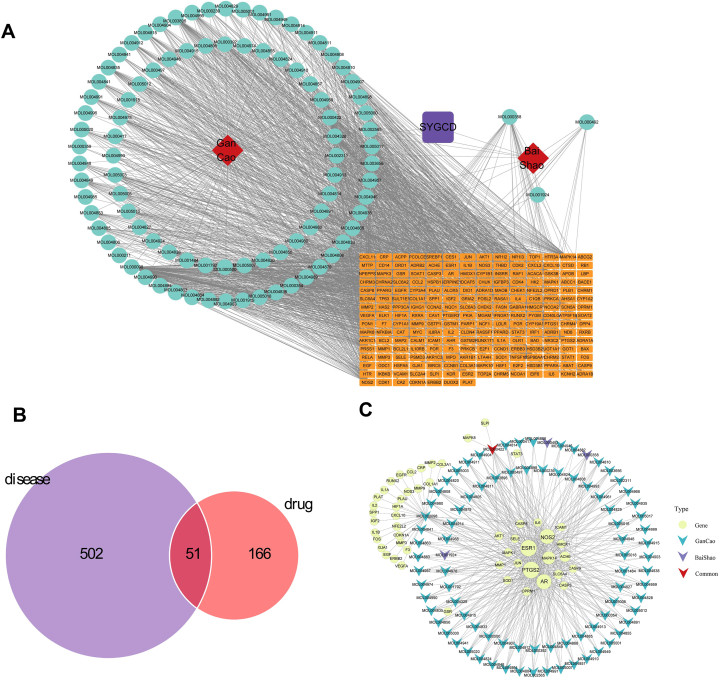
Identification of potential protein targets of SGD formula. (A) A total of 93 active ingredients were obtained from SGD formula using TCMSP, BATMAN-TCM, and TCMID databases. The interaction network shows a total of 217 corresponding protein targets for the 93 active ingredients. (B) Venn diagram showing 51 common targets between 553 LDD-related genes and 217 protein targets of SGD formula. (C) The 51 overlapping targets corresponds 87 active components from SGD formula.

The active ingredients of SGD formula were retrieved from TCMSP, BATMAN-TCM, and TCMID databases. A total of 93 active ingredients were obtained, among which 3 compounds are derived from Shaoyao, 89 are derived from Gancao and 1 ingredient is shared by Shaoyao and Gancao. Subsequently, a total of 217 corresponding protein targets were identified using these active components (Fig. 1A). Next, 553 gene targets associated with LDD were retrieved from genecard, OMIM, and TTD databases. These disease targets were then compared with drug targets to identify 51 potential drug-related disease targets for further investigation (Fig. 1B). These 51 protein targets correspond to 87 active components from SGD formula (Fig. 1C).

**Fig. 2:**
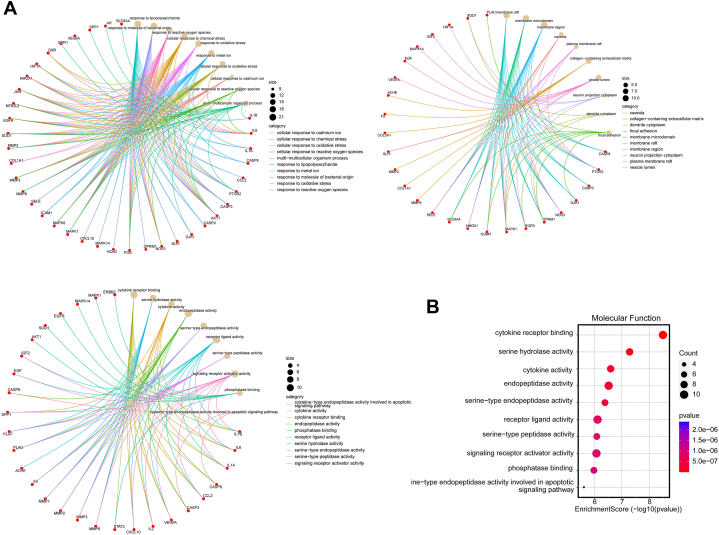
Enrichment analysis of the candidate targets. (A) The top 5 hits of 1403 biological processes (BP), 27 cellular components (CC) and 77 molecular functions (MF) enriched in the candidate targets. (B) The top 10 ranked KEGG pathways.

To gain the biological insights of the candidate targets, GO and KEGG enrichment analysis was performed. There were 1403 biological processes (BP), 27 cellular components (CC) and 77 molecular functions (MF) enriched in the candidate targets (Fig. 2A, top 5 hits were displayed), along with 136 KEGG pathways (Fig. 2B, top 10 ranked pathways were shown). These results suggest that the candidate targets are implicated in the inflammatory pathway.

### 3.2 Screening of the core targets in LDD

**Fig. 3:**
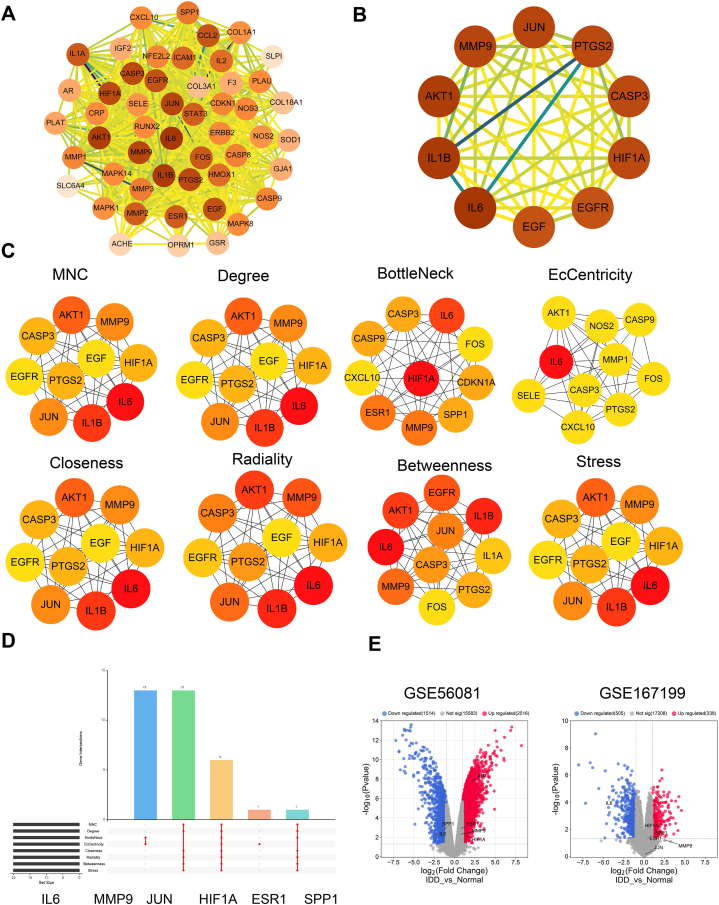
Interrogation of the core targets in the PPI network. The protein-protein interaction (PPI) network of the 51 drug-related disease targets, which consists of 51 nodes and 782 edges. (B) The top 10 nodes in the network. (C) The ranking of core targets in the network using various analysis modules (MNC, Degree, BottleNeck, EcCentricity, Closeness, Radiality, Betweenness, and Stress) in cytohubba. (D) The top 20 targets of the above-mentioned scoring modules were integrated to get 6 core targets: IL6, MMP9, JUN, HIF1A, ESR1 and SPP1. (E) Volcano blot showing the differentially expressed genes in LDD samples from GSE56081 and GSE167119 dataset.

The protein-protein interaction (PPI) network was constructed based on the 51 drug-related disease targets, which consists of 51 nodes and 782 edges (Fig. 3A). The node degree values were calculated using the cytoNCA plug-in, with darker colors indicating higher degrees. The top 10 nodes in the network are IL6, IL1B, Akt1, Jun, MMP9, PTGS2, CASP3, HIF1A, EGFR, and EGF (Fig. 3B). To assess the core targets in the network, we utilized various analysis modules (MNC, Degree, BottleNeck, EcCentricity, Closeness, Radiality, Betweenness, and Stress) available in cytohubba, a built-in function of Cytoscape software. These modules were used to score the target proteins, and each module identified slightly different top 10 core nodes in the PPI network (Fig. 3C). Then, the top 20 targets of the above-mentioned scoring modules were integrated to get 6 core targets: IL6, MMP9, JUN, HIF1A, ESR1 and SPP1 (Fig. 3D). We next sought to examine the expression pattern of these targets in LDD samples. Through the analysis of published microarray datasets, we found that SPP1 and IL6 were significantly down-regulated, while JUN and HIF1A showed up-regulation in the LDD samples of the GSE56081 dataset. Similarly, in the GSE167119 dataset, IL6 was significantly down-regulated, while SPP1 and HIF1A showed up-regulation in the LDD samples (Fig. 3E). Therefore, IL6 and HIF1A are selected as the final candidates for further analysis, since they showed consistent expression changes in two independent datasets.

**Fig. 4:**
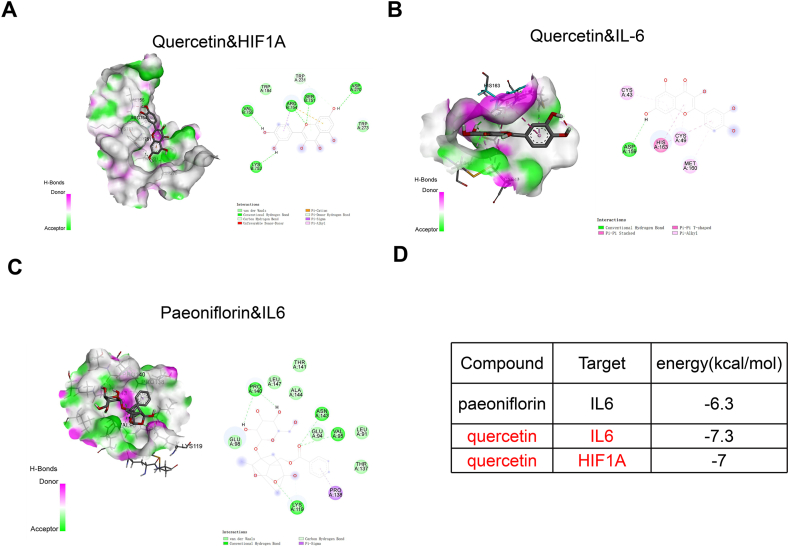
Molecular docking analysis Molecular docking results revealed (A) the interaction between HIF1A and quercetin, (B) the interaction between IL6 with paeoniflorin, and (C) the interaction between IL6 and quercetin. (D) Summary of the optimal model binding energy and the interaction force between the docking area of each pair.

Molecular docking indicated the interactions between HIF1A and quercetin, and IL6 with paeoniflorin and quercetin (Fig. 4A–C). In all the predicted pairs, the number of hydrogen bonds is greater than 2. Further, the analysis of optimal model binding energy and interaction force between the docking area showed that the binding energy is less than −5.0 kcal/mol (Fig. 4D). These data strongly support the interaction between HIF1A and quercetin, as well as the interactions between IL6 with paeoniflorin/quercetin.

### 3.3 A cell pressure model of LDD reveals the up-regulation of HIF1A and down-regulation of IL6

**Fig. 5:**
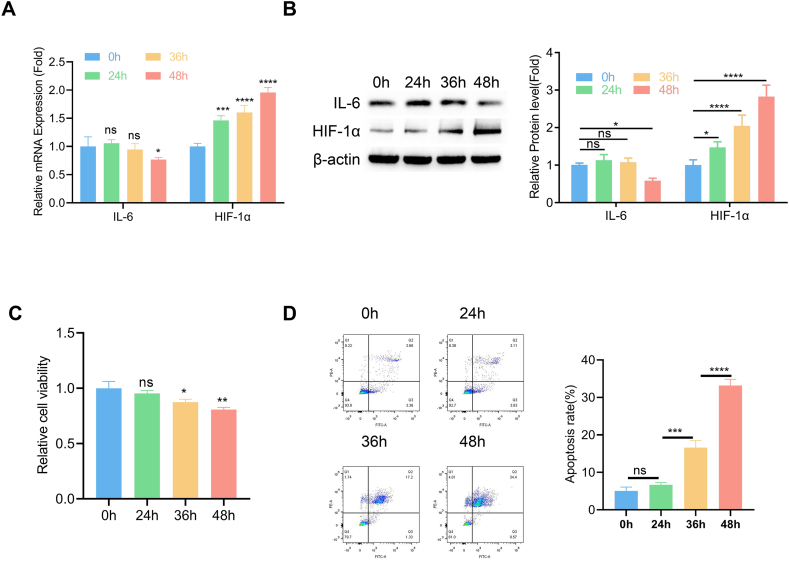
A cell pressure model reveals the up-regulation of HIF1A and down-regulation of IL6 A cell pressure model of nucleus pulposus mesenchymal stem cells (NPMSCs) was established by inducing 1.0 MPa compression for different duration. (A) qRT-PCR analysis of HIF1A and IL6 mRNA after different duration of compression. (B) Immunoblotting of HIF1A and IL6 protein after different duration of compression. (C) Cell viability examination by CCK-8 assay. (D) Apoptotic events were quantified by flow cymotetry method. N = 3 experiments. One-way ANOVA test was adopted with Tukey's post hoc test. *p < 0.05; **p < 0.01; ***p < 0.001; ****p < 0.0001.

**Fig. S1:**
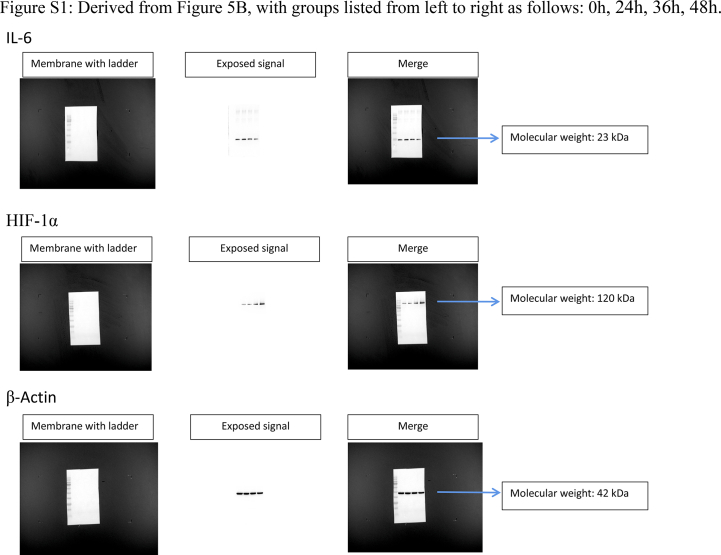
Original uncropped versions of blotting images for data in Fig. 5B.

To simulate the compression in LDD condition, a cell pressure model of nucleus pulposus mesenchymal stem cells (NPMSCs) was established by inducing 1.0 MPa compression for different duration (24, 36, and 48 h). We observed a dramatic increase of HIF1A expression and a down-regulation of IL6 at both the protein and mRNA levels after 48 h of pressure culture (Fig. 5A–B, Fig. S1). Furthermore, continuous application of compression impaired cell viability and increased apoptotic cell death in NPMSCs (Fig. 5C–D). These results confirmed the expression changes of HIF1A and IL6 in the cell pressure model, which is consistent with the data of transcriptome analysis in the LDD samples.

### 3.4 Quercetin alleviates pressure-induced cell death in NPMSCs

**Fig. 6:**
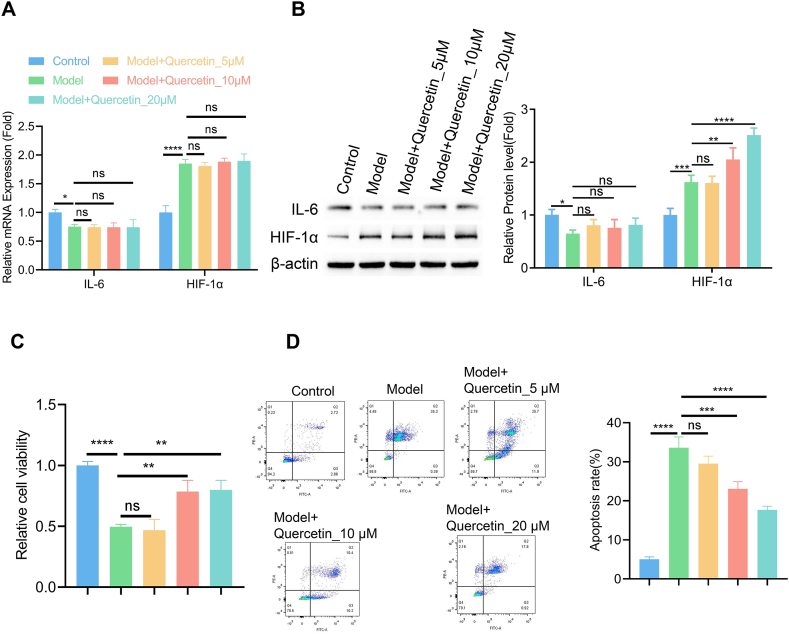
Quercetin alleviates pressure-induced cell death in NPMSCs In the cell pressure model for 48 h induction, quercetin was applied at 5–20 μM. (A) The mRNA levels of IL6 and HIF1A were quantified by qRT-PCR. (B) The protein levels of IL6 and HIF1A were analyzed by immunoblotting. (C) Cell viability examination by CCK-8 assay. (D) Apoptotic events were quantified by flow cymotetry method. N = 3 experiments. One-way ANOVA test was adopted with Tukey's post hoc test. *p < 0.05; **p < 0.01; ***p < 0.001; ****p < 0.0001.

**Fig. S2:**
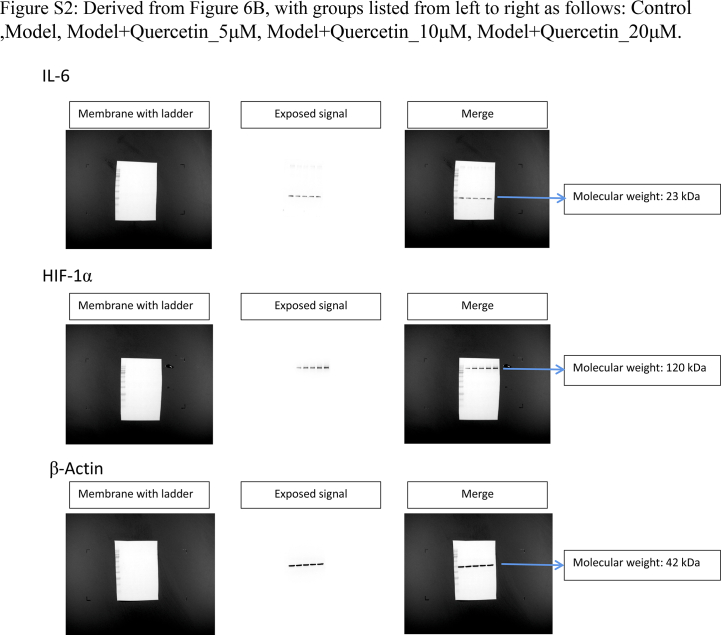
Original uncropped versions of blotting images for data in Fig. 6B.

Since HIF1A showed dramatic changes in the cell model starting from 24 h induction, while IL6 only display a mild decrease after 48 h (Fig. 5A and B), we selected HIF1A as the target for the following study and applied the bioactive compound from SGS (quercetin) which could potentially interact with HIF1A. In the cell pressure model for 48 h induction, the application of quercetin at 5–20 μM did not alter the mRNA levels of both IL6 and HIF1A (Fig. 6A). However, the treatment of quercetin at 10 and 20 μM increased the protein expression of HIF1A (Fig. 6B, Fig. S2). Meanwhile, quercetin treatment at 10 and 20 μM promoted cell survival and inhibited apoptosis under the compression condition (Fig. 6C and D). These results imply that quercetin could alleviate pressure-induced cell death in NPMSCs, possibly through stabilizing HIF1A protein.

### 3.5 Quercetin stabilizes HIF1A protein in NPMSCs

**Fig. 7:**
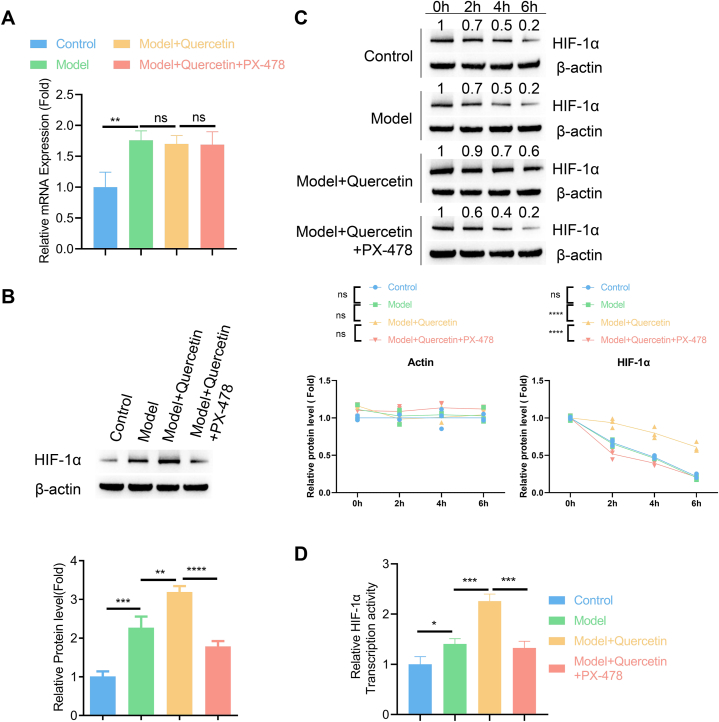
Quercetin stabilizes HIF1A protein in NPMSCs NPMSCs under the compression were treated with 20 μM quercetin in the presence or absence of 5 μM PX-478 (an HIF1A inhibitor). (A) The mRNA levels of HIF1A were quantified by qRT-PCR. (B) The protein levels of HIF1A were analyzed by immunoblotting. (C) Cycloheximide (CHX) was applied to block *de novo* protein synthesis in above conditions, and the remaining protein levels of HIF1A at different time points after CHX addition were analyzed by immunoblotting, with actin serving as a internal control. (D) Quantification of transcription activity of HIF1A in above experimental conditions. N = 3 experiments. One-way ANOVA test was adopted with Tukey's post hoc test in A, B, and D. Two-way ANOVA test was adopted with Tukey's post hoc test in C. *p < 0.05; **p < 0.01; ***p < 0.001; ****p < 0.0001.

**Fig. S3:**
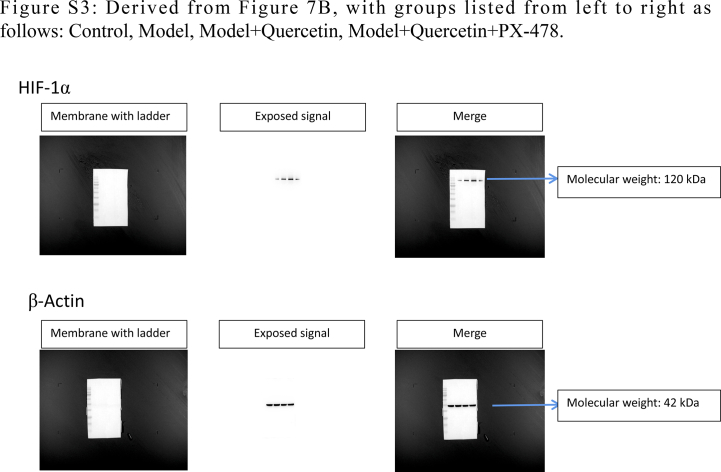
Original uncropped versions of blotting images for data in Fig. 7B.

**Fig. S4:**
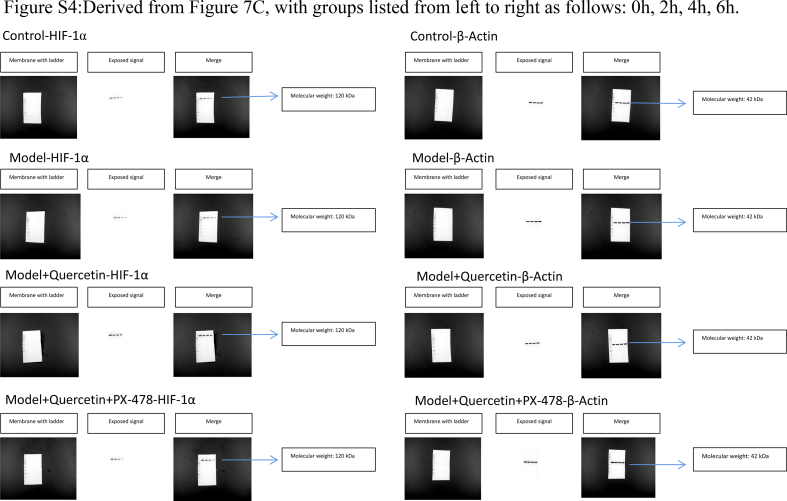
Original uncropped versions of blotting images for data in Fig. 7C.

To corroborate the effect of quercetin effect on HIF1A stability, cells under the compression were treated with 20 μM quercetin in the presence or absence of 5 μM PX-478 (an HIF1A inhibitor). These treatments did not affect the mRNA levels of HIF1A under pressure condition (Fig. 7A). Quercetin treatment increased HIF1A protein level, and this effect was abrogated by PX-478 (Fig. 7B, Fig. S3). We next applied cycloheximide (CHX) to block *de novo* protein synthesis in above conditions, and collected protein samples at different time points after CHX addition to quantify the stability of HIF1A. In the control and compression model cells, HIF1A levels decreased dramatically after CHX treatment. Contrarily, in the experimental group with quercetin treatment, HIF1A protein levels decreased to a less extent at each time point and this effect of quercetin was largely abolished by PX-478 (Fig. 7C, Fig. S4). Therefore, these data demonstrate the stabilizing effect of quercetin treatment on HIF1A protein. We also examined the transcription activity of HIF1A in NPMSCs. Compared to the control, cells in compression model group displayed an elevated transcription activity of HIF1A, and quercetin treatment further increased HIF1A activity. The application of PX-478 blocked the effect of quercetin treatment (Fig. 7D). These data are in agreement with the changes of HIF1A protein levels in each condition.

### 3.6 The rescue effect of quercetin on pressure-induced oxidative stress and cell death depends on HIF1A protein

**Fig. 8:**
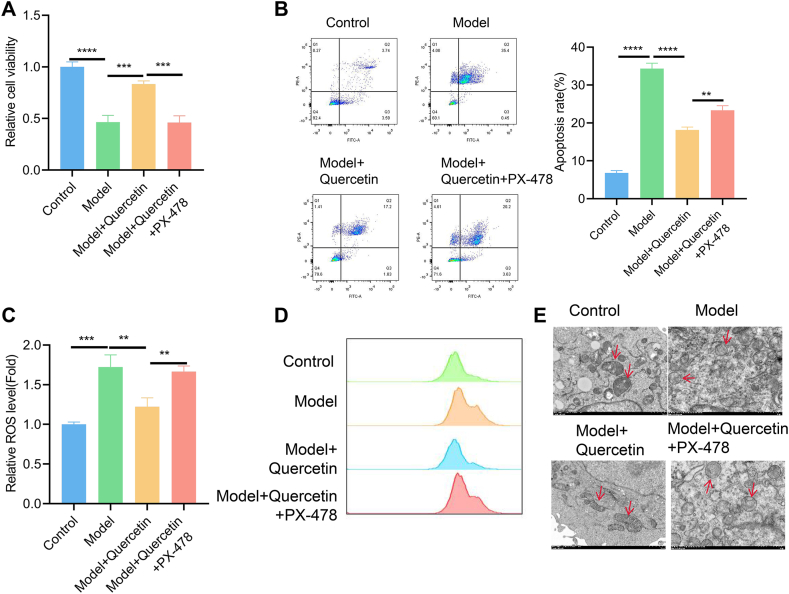
The rescue effect of quercetin on pressure-induced oxidative stress and cell death depends on HIF1A protein NPMSCs under the compression were treated with 20 μM quercetin in the presence or absence of 5 μM PX-478 (an HIF1A inhibitor). (A) Cell viability examination by CCK-8 assay. (B) Apoptotic events were quantified by flow cymotetry method. (C) The measurement of cellular levels of reactive oxygen species (ROS) and (D) mitochondria-derived ROS. (E) Electron microscope analysis of mitochondrial phenotype, and the arrows indicates the changes of mitochondrial cirstea. N = 3 experiments. One-way ANOVA test was adopted with Tukey's post hoc test. *p < 0.05; **p < 0.01; ***p < 0.001; ****p < 0.0001.

We next investigated whether HIF1A inhibition abolishes the rescue effect of quercetin on pressure-induced cell death. Cell viability and apoptosis measurement demonstrated that the presence of HIF1A inhibitor PX-478 largely abrogated the protective effect of quercetin on pressure-induced cell death (Fig. 8A and B). We also observed that the cellular levels of reactive oxygen species (ROS) and mitochondria-derived ROS were elevated in pressure-induced cells. The elevation of oxidative stress was alleviated by quercetin, but HIF1A inhibition exacerbated the oxidative burden (Fig. 8C and D). Electron microscope analysis revealed a disruption of mitochondrial cirstea under compression condition. Quercetin treatment rescued mitochondrial phenotype while this effect was abolished by HIF1A inhibition (Fig. 8E). These findings suggest that the rescue effect of quercetin on pressure-induced oxidative stress and cell death depends on HIF1A protein.

### 3.7 Quercetin ameliorates LDD in the rat model

**Fig. 9:**
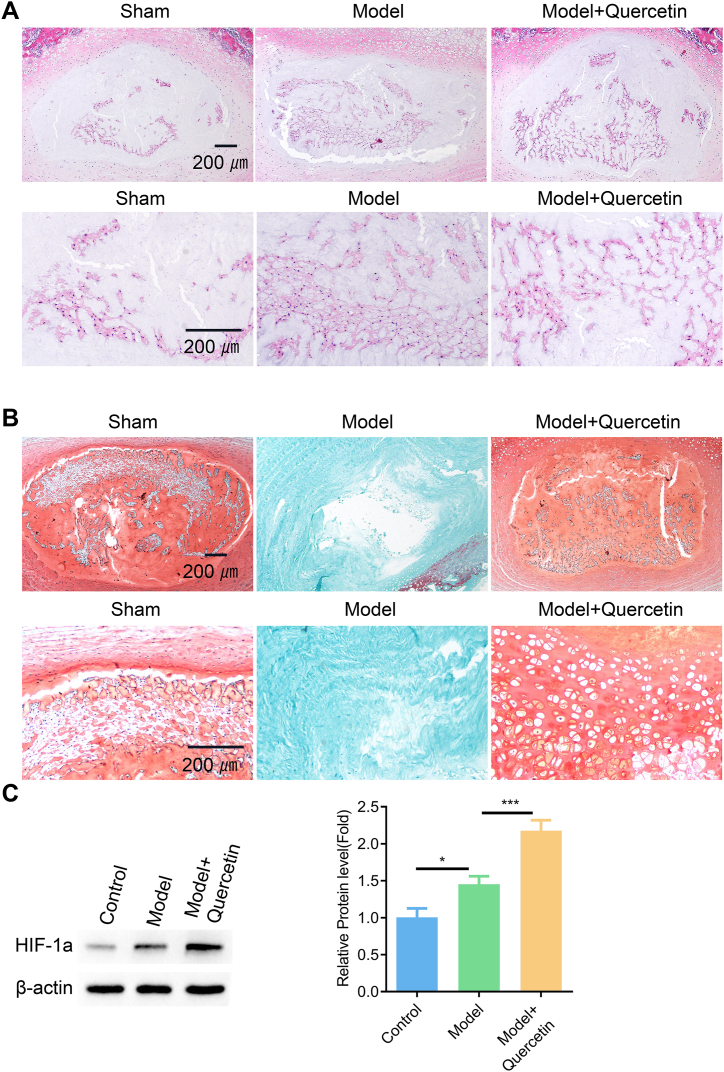
Quercetin ameliorates LDD in the rat model A rat model of compression-induced LDD was induced for 4 weeks, and quercetin was administrated for 2 weeks in the drug intervention group after LDD induction. **(**A) H&E and (B) Safranin O-fast Green staining in intervertebral disc tissues. (C) Western blot analysis of HIF1A expression in intervertebral disc samples. N = 6 animals in each group. One-way ANOVA test was adopted with Tukey's post hoc test. *p < 0.05; **p < 0.01; ***p < 0.001; ****p < 0.0001.

**Fig. S5:**
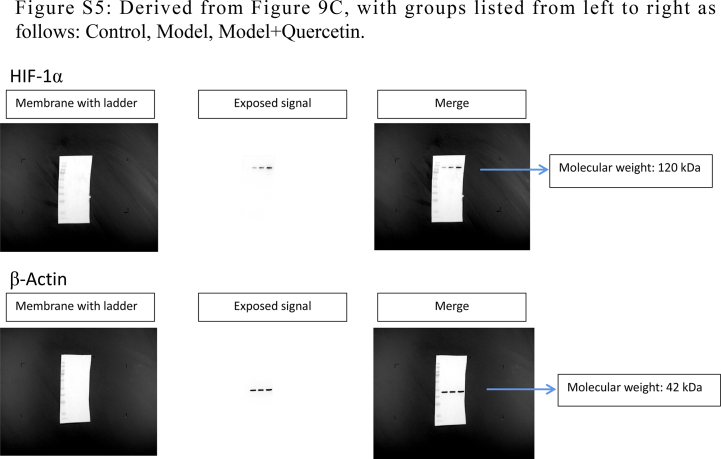
Original uncropped versions of blotting images for data in Fig. 9C.

To further evaluate the potential protective effect of quercetin on LDD, we constructed a rat model of compression-induced LDD. H&E and Safranin O-fast Green staining revealed the disruption of intervertebral disc structure and a reduced level of cartilage in compression-induced LDD model group. Quercetin treatment significantly restored the structure of intervertebral disc structure and improved cartilage level, thus attenuating the deteriorating effect of compression (Fig. 9A and B). The analysis of HIF1A expression by immunoblotting demonstrated an increased HIF1A protein level in intervertebral disc after compression, and quercetin treatment further enhanced the protein expression of HIF1A (Fig. 9C, Fig. S5). Taken together, our findings suggest that compression induces HIF1A up-regulation in intervertebral disc, which may serve as a protective mechanism in LDD. Quercetin treatment further stabilizes HIF1A protein level to promote the recovery from compression-induced damages.

## 4 Discussion

In the current investigation, the potential protein targets of SGD formula were identified and HIF1A was selected as a target of quercetin for further study in LDD. We demonstrated that HIF1A was up-regulated in the LDD samples and compression-induced NPMSCs. Quercetin alleviated pressure-induced cell death in NPMSCs by stabilizing HIF1A. The protective effect of quercetin on pressure-induced oxidative stress and cell death was abrogated upon HIF1A inhibition. Quercetin also ameliorated LDD in the rat model. Altogether, our findings pinpoint quercetin as a bioactive compound from SGD formula, which could protect NPMSCs from compression-induced cell death.

Numerous factors have been identified to play a role in the development of LDD, including age, metabolic processes, dietary intake, and physical strain [36,37]. Of all these detrimental factors, overloading the spine mechanically has been pinpointed as a primary instigator of LDD [38]. Furthermore, the fundamental workings behind compression-induced LDD have been extensively elucidated, with numerous signaling pathways shown to play a role in its progression, such as the TGF-β pathway and the PI3K/Akt pathway [36,39]. The precursor cells found within the intervertebral disc, consisting of cartilage endplate progenitor cells and NPMSCs, have been identified as key cellular components to facilitate the regeneration of the intervertebral disc [7,8]. Here, we showed that compression imposed on NPMSCs resulted in oxidative stress and triggered apoptosis, which is in agreement with the previous studies [40,41]. Thus, alleviating oxidative stress induced apoptosis can be an intervention to suppress LDD.

Shaoyao-Gancao decoction (SGD) is a traditional Chinese medicinal formula with demonstrated antioxidant and anti-apoptotic effects [[12], [13], [14],^42^,^43^]. For instance, SGD administration suppressed oxidative stress and apoptosis in ethanol-induced gastric ulcers [43]. Through network pharmacology analysis, we revealed quercetin as a key bioactive component from SGD to target LDD. We showed that quercetin was able to suppress compression-induced apoptosis and protect against mitochondria-derived ROS in NPMSCs. Further, quercetin administration alleviated LDD progression in compression induced rat model. Quercetin is a potent antioxidant flavonoid, and its beneficial effects were widely reported in different pathophysiological conditions [44]. The anti-inflammatory and antioxidant properties makes quercetin an attractive therapeutic agent in cardiovascular and neurodegenerative disorders [[45], [46], [47]]. Mechanistic studies in different models revealed that quercetin defense against oxidative burden by promoting mitochondrial biogenesis and fitness [[48], [49], [50]]. Consistent with this, our study suggest that quercetin preserves mitochondrial function under compression, thereby alleviating ROS production and protecting against apoptosis in NPMSCs. These findings are also in agreement with the notion that mitochondrial quality control is a key adaptation to avoid oxidative damages [51,52].

Hypoxia-inducible factor 1 (HIF-1) orchestrates cellular adaptation to the deprivation of oxygen by regulating gene expression. HIF1A has a close relationship with mitochondrial functions [[53], [54]]. There is compelling evidence that HIF1A mediates adaptation to hypoxia by suppressing mitochondrial oxygen consumption, thus alleviating ROS generation and apoptosis [55,56]. In response to oxidative stress, HIF1A is transported to the mitochondria to shield against apoptosis triggered by oxidative stress. Mitochondrial HIF-1α decreases levels of ROS and mitigate damage to the mitochondria [57]. Furthermore, HIF1A could relieve high-glucose-induced oxidative damages in renal cells by promoting mitophagy [58]. Our data showed an up-regulation of HIF1A in the LDD tissues and compression-induced NPMSCs, which may be an intrinsic mechanism to fight against oxidative stress. These data are in consistence with a previous report that HIF1A is induced under compression stress [59]. We further demonstrated that quercetin suppressed compression-induced cell death and mitochondrial ROS production in NPMSCs by stabilizing HIF1A, an effect which was abolished upon HIF1A inhibition. In line wit this, previous studies revealed that HIF1A activation could alleviate compression-induced apoptosis in stem cells [60,61]. Furthermore, previous studies also highlighted the activation of HIF1A signaling by quercetin in different types of cells [[62], [63], [64], [65]]. These evidence and our findings altogether imply that quercetin protects against compression-induced LDD by stabilizing HIF1A.

While the results identify HIF1A as a potential therapeutic target of quercetin in alleviating compression-induced cell death and disc degeneration, there are some important limitations to consider. The *in vitro* cell culture models and rat model utilized may not fully recapitulate the complex pathophysiology of human lumbar disc degeneration. Additionally, the study focused primarily on the HIF1A pathway, but LDD likely involves dysregulation of multiple signaling mechanisms. Future studies should aim to validate these findings in larger animal models that better reflect human disc anatomy and biomechanics. Comprehensive investigations into the synergistic effects of multiple bioactive components from the SGD formula are also warranted, as quercetin alone may not recapitulate the full therapeutic potential. Furthermore, evaluating the pharmacokinetic profiles, bioavailability, and long-term safety of quercetin or the herbal formula will be crucial for translation into clinical applications. Ultimately, well-designed clinical trials are needed to assess the efficacy and safety of these potential therapeutic agents in human patients with LDD.

## 5 Conclusion

To sum up, our study identified HIF1A as a key protective factor against compression-induced death of NPMSCs, a pivotal cell type implicated in LDD. Quercetin, a bioactive compound from the traditional Chinese medicine SGD, alleviated compression-induced oxidative stress, apoptosis and cell death in NPMSCs by stabilizing HIF1A protein. Importantly, in a rat model of LDD, quercetin treatment ameliorated disease progression. These findings uncover quercetin as a novel HIF1A-stabilizing agent that can preserve NPMSC survival and combat LDD. Targeting the HIF1A pathway represents a promising therapeutic strategy for degenerative disc diseases. Further research evaluating the long-term effects and clinical translation of quercetin or other HIF1A modulators could potentially lead to efficacious, disease-modifying treatments for the debilitating chronic low back pain caused by disc degeneration.

## Ethics approval and consent to participate

The use of human samples gained the approval of the medical research ethics committee of the Medical Ethics Committee of The 920th Hospital of Joint Logistics SupportForce of PLA (Lunshen 2024-005(Ke)-01). All the recruited subjects signed the written informed consent. All the sample handling and data processing steps were following the Declaration of Helsinki.

Animal protocols were in compliance with the guidelines of animal care and welfare and were approved by The 920th Hospital of Joint Logistics SupportForce of PLA (Lunshen 2024-005(Ke)-01).

## Consent for publication

All authors are aware of and have consented to the publication.

## Funding

This work was supported by Yunnan Orthopedics and sports rehabilitation Clinical Medical Research Center (202102AA310068); Trauma Orthopaedic Clinical medical center of Yunnan Province (ZX20191001); Yunnan Province technology innovation talent training object project (202005AD160146).

## Availability of data and materials

The data generated in this study are available upon request to the corresponding author.

## CRediT authorship contribution statement

**Junxiao Ren:** Writing – original draft, Funding acquisition, Data curation. **Rui Xin:** Writing – original draft, Project administration. **Xiaoping Cui:** Validation, Formal analysis. **Yongqing Xu:** Visualization, Methodology, Investigation. **Chuan Li:** Writing – review & editing, Conceptualization.

## Declaration of competing interest

The authors declare that they have no known competing financial interests or personal relationships that could have appeared to influence the work reported in this paper.

## References

[1] Costăchescu B., Niculescu A.G., Teleanu R.I., Iliescu B.F., Rădulescu M., Grumezescu A.M., Dabija M.G. (2022 Jun 9). Recent advances in managing spinal intervertebral discs degeneration. Int. J. Mol. Sci..

[2] Ravindra V.M., Senglaub S.S., Rattani A., Dewan M.C., Härtl R., Bisson E., Park K.B., Shrime M.G. (2018 Dec). Degenerative lumbar spine disease: estimating global incidence and worldwide volume. Global Spine J..

[3] Hanaei S., Abdollahzade S., Khoshnevisan A., Kepler C.K., Rezaei N. (2015). Genetic aspects of intervertebral disc degeneration. Rev. Neurosci..

[4] Xiao L., Majumdar R., Dai J., Li Y., Xie L., Shen F.H., Jin L., Li X. (2019 Apr 8). Molecular detection and assessment of intervertebral disc degeneration via a collagen hybridizing peptide. ACS Biomater. Sci. Eng..

[5] Ding S.L., Zhang T.W., Zhang Q.C., Ding W., Li Z.F., Han G.J., Bai J.S., Li X.L., Dong J., Wang H.R., Jiang L.B. (2021 Dec). Excessive mechanical strain accelerates intervertebral disc degeneration by disrupting intrinsic circadian rhythm. Exp. Mol. Med..

[6] Fan H., Chen Z., Tang H.B., Shan L.Q., Chen Z.Y., Liu S.C., Zhang Y.Y., Guo X.Y., Yang H., Hao D.J. (2022 Sep 21). Necroptosis of nucleus pulposus cells involved in intervertebral disc degeneration through MyD88 signaling. Front. Endocrinol..

[7] Liu Y., Li Y., Huang Z.N., Wang Z.Y., Nan L.P., Wang F., Zhou S.F., Wang J.C., Feng X.M., Zhang L. (2019 Jul). The effect of intervertebral disc degenerative change on biological characteristics of nucleus pulposus mesenchymal stem cell: an in vitro study in rats. Connect. Tissue Res..

[8] Jia Z., Yang P., Wu Y., Tang Y., Zhao Y., Wu J., Wang D., He Q., Ruan D. (2017 Jun). Comparison of biological characteristics of nucleus pulposus mesenchymal stem cells derived from non-degenerative and degenerative human nucleus pulposus. Exp. Ther. Med..

[9] Ou X., Ying J., Bai X., Wang C., Ruan D. (2020 Sep). Activation of SIRT1 promotes cartilage differentiation and reduces apoptosis of nucleus pulposus mesenchymal stem cells via the MCP1/CCR2 axis in subjects with intervertebral disc degeneration. Int. J. Mol. Med..

[10] Yurube T., Takeoka Y., Kanda Y., Kuroda R., Kakutani K. (2023 Mar 11). Intervertebral disc cell fate during aging and degeneration: apoptosis, senescence, and autophagy. N Am Spine Soc J.

[11] Zhang Y.Y., Hu Z.L., Qi Y.H., Li H.Y., Chang X., Gao X.X., Liu C.H., Li Y.Y., Lou J.H., Zhai Y., Li C.Q. (2022 Jul 26). Pretreatment of nucleus pulposus mesenchymal stem cells with appropriate concentration of H2O2 enhances their ability to treat intervertebral disc degeneration. Stem Cell Res. Ther..

[12] Bi X., Gong M., Di L. (2014). Review on prescription compatibility of shaoyao gancao decoction and reflection on pharmacokinetic compatibility mechanism of traditional Chinese medicine prescription based on in vivo drug interaction of main efficacious components. Evid Based Complement Alternat Med.

[13] Sun L., Zhao M., Li J., Liu J., Wang M., Zhao C. (2023 Apr). Exploration of the anti-liver injury active components of Shaoyao Gancao decoction by network pharmacology and experiments in vivo. Phytomedicine.

[14] Luo Y., Qiu Y., Zhou R., Zhang Y., Ji X., Liu Z., Li R., Zhang Y., Yang F., Hou J., Zhang S., Wang T., Song H., Tao X. (2023 Dec 5). Shaoyao Gancao decoction alleviates the central hyperalgesia of recurrent NTG-induced migraine in rats by regulating the NGF/TRPV1/COX-2 signal pathway. J. Ethnopharmacol..

[15] Zhu G.Y., Jia D.D., Yang Y., Miao Y., Wang C., Wang C.M. (2021 Sep 15). The effect of shaoyao gancao decoction on sphincter of oddi dysfunction in hypercholesterolemic rabbits via protecting the enteric nervous system-interstitial cells of cajal-smooth muscle cells network. J. Inflamm. Res..

[16] Chiu Y.J., Lin C.H., Lee M.C., Hsieh-Li H.M., Chen C.M., Wu Y.R., Chang K.H., Lee-Chen G.J. (2021 Jun 9). Formulated Chinese medicine Shaoyao Gancao Tang reduces NLRP1 and NLRP3 in Alzheimer's disease cell and mouse models for neuroprotection and cognitive improvement. Aging (Albany NY).

[17] Chang Z.P., Deng G.F., Shao Y.Y., Xu D., Zhao Y.N., Sun Y.F., Zhang S.Q., Hou R.G., Liu J.J. (2021 May 13). Shaoyao-Gancao decoction ameliorates the inflammation state in polycystic ovary syndrome rats via remodeling gut microbiota and suppressing the TLR4/NF-κB pathway. Front. Pharmacol..

[18] Xu Y., Li C., Chen T., Li X., Wu X., Zhang Q., Zhao L. (2022 Dec 2). Quantitative analysis of the multicomponent and spectrum-effect correlation of the antispasmodic activity of shaoyao-gancao decoction. J Anal Methods Chem..

[19] Chen I.C., Lin T.H., Hsieh Y.H., Chao C.Y., Wu Y.R., Chang K.H., Lee M.C., Lee-Chen G.J., Chen C.M. (2018 Oct 28). Formulated Chinese medicine shaoyao gancao tang reduces tau aggregation and exerts neuroprotection through anti-oxidation and anti-inflammation. Oxid. Med. Cell. Longev..

[20] Chen C.M., Chen W.L., Hung C.T., Lin T.H., Lee M.C., Chen I.C., Lin C.H., Chao C.Y., Wu Y.R., Chang K.H., Hsieh-Li H.M., Lee-Chen G.J. (2019 Feb 13). Shaoyao Gancao Tang (SG-Tang), a formulated Chinese medicine, reduces aggregation and exerts neuroprotection in spinocerebellar ataxia type 17 (SCA17) cell and mouse models. Aging (Albany NY).

[21] Wang P., Yin Q.W., Zhang A.H., Sun H., Wu X.H., Wang X.J. (2014 Oct). Preliminary identification of the absorbed bioactive components and metabolites in rat plasma after oral administration of Shaoyao-Gancao decoction by ultra-performance liquid chromatography with electrospray ionization tandem mass spectrometry. Pharmacogn Mag.

[22] Wang Y., Cheng H., Wang T., Zhang K., Zhang Y., Kang X. (2023 Sep). Oxidative stress in intervertebral disc degeneration: molecular mechanisms, pathogenesis and treatment. Cell Prolif..

[23] Yurube T., Takeoka Y., Kanda Y., Kuroda R., Kakutani K. (2023 Mar 11). Intervertebral disc cell fate during aging and degeneration: apoptosis, senescence, and autophagy. N Am Spine Soc J.

[24] Li Z., Chen S., Ma K., He R., Xiong L., Hu Y., Deng X., Yang A., Ma X., Shao Z. (2020 Sep). Comparison of different methods for the isolation and purification of rat nucleus pulposus-derived mesenchymal stem cells. Connect. Tissue Res..

[25] Onal S., Alkaisi M.M., Nock V. (2022 Nov 9). Microdevice-based mechanical compression on living cells. iScience.

[26] Rao X., Huang X., Zhou Z., Lin X. (2013 Aug). An improvement of the 2^(-delta delta CT) method for quantitative real-time polymerase chain reaction data analysis. Biostat Bioinforma Biomath.

[27] Yang X., Zhong Y., Wang D., Lu Z. (2021 Nov 11). A simple colorimetric method for viable bacteria detection based on cell counting Kit-8. Anal. Methods.

[28] Yang Y., Zhang G., Yang T., Gan J., Xu L., Yang H. (2021 Apr 20). A flow-cytometry-based protocol for detection of mitochondrial ROS production under hypoxia. STAR Protoc..

[29] Xie X., Endara-Coll M., Mahmood R., Jankauskas R., Gjorgjieva T., Percipalle P. (2019 Oct). Mitochondria-localized β-actin is essential for priming innate antiviral immune signaling by regulating IRF3 protein stability. Cell. Mol. Immunol..

[30] Jeknić S., Kudo T., Song J.J., Covert M.W. (2023 Apr). An optimized reporter of the transcription factor hypoxia-inducible factor 1α reveals complex HIF-1α activation dynamics in single cells. J. Biol. Chem..

[31] Ng N.S., Ooi L. (2021 Jan 5). A simple microplate assay for reactive oxygen species generation and rapid cellular protein normalization. Bio Protoc.

[32] Ngo L., Nathanson A.D., Garbowski T., Knothe U., Zeidler D., Knothe Tate M.L. (2019 Jul 20). Electron microscopy sample preparation protocol enabling nano-to-mesoscopic mapping of cellular connectomes and their habitats in human tissues and organs. Bio Protoc..

[33] Xue F., Wei Y., Chen Y., Wang Y., Gao L. (2014 Feb). A rat model for chronic spinal nerve root compression. Eur. Spine J..

[34] Chen W.J., Cheng Y., Li W., Dong X.K., Wei J.L., Yang C.H., Jiang Y.H. (2021 Oct 28). Quercetin attenuates cardiac hypertrophy by inhibiting mitochondrial dysfunction through SIRT3/PARP-1 pathway. Front. Pharmacol..

[35] Kalender Y., Kaya S., Durak D., Uzun F.G., Demir F. (2012 Mar). Protective effects of catechin and quercetin on antioxidant status, lipid peroxidation and testis-histoarchitecture induced by chlorpyrifos in male rats. Environ. Toxicol. Pharmacol..

[36] Bian Q., Ma L., Jain A., Crane J.L., Kebaish K., Wan M., Zhang Z., Edward Guo X., Sponseller P.D., Séguin C.A., Riley L.H., Wang Y., Cao X. (2017 Mar 21). Mechanosignaling activation of TGFβ maintains intervertebral disc homeostasis. Bone Res..

[37] Kirnaz S., Capadona C., Wong T., Goldberg J.L., Medary B., Sommer F., McGrath L.B., Härtl R. (2022 Jan). Fundamentals of intervertebral disc degeneration. World Neurosurg.

[38] Adams M.A., Freeman B.J., Morrison H.P., Nelson I.W., Dolan P. (2000 Jul 1). Mechanical initiation of intervertebral disc degeneration. Spine.

[39] Li P., Liang Z., Hou G., Song L., Zhang R., Gan Y., Zhang C., Xu Y., Zhou Q. (2017). N-Cadherin-Mediated activation of PI3K/Akt-GSK-3β signaling attenuates nucleus pulposus cell apoptosis under high-magnitude compression. Cell. Physiol. Biochem..

[40] Ma K.G., Shao Z.W., Yang S.H., Wang J., Wang B.C., Xiong L.M., Wu Q., Chen S.F. (2013 Dec). Autophagy is activated in compression-induced cell degeneration and is mediated by reactive oxygen species in nucleus pulposus cells exposed to compression. Osteoarthritis Cartilage.

[41] Huang D., Peng Y., Ma K., Qing X., Deng X., Li Z., Shao Z. (2020 Jan 11). Puerarin relieved compression-induced apoptosis and mitochondrial dysfunction in human nucleus pulposus mesenchymal stem cells via the PI3K/Akt pathway. Stem Cell. Int..

[42] Zeng Y.Y., Li K.Y. (2017). Effects of jiawei shaoyao-gancao decoction and its drug-containing serum on proliferation, apoptosis, and ultrastructure of human adenomyosis foci cells. Evid Based Complement Alternat Med.

[43] Jin Y., Zhang M., Wang Y., Lu Y., Liu T., Yang G., Song S., Liu W. (2022 Apr 11). Protective effect and potential mechanism of the traditional Chinese medicine shaoyao-gancao decoction on ethanol-induced gastric ulcers in rats. Evid Based Complement Alternat Med.

[44] Anand David A.V., Arulmoli R., Parasuraman S. (2016 Jul-Dec). Overviews of biological importance of quercetin: a bioactive flavonoid. Pharmacogn Rev.

[45] Xu D., Hu M.J., Wang Y.Q., Cui Y.L. (2019 Mar 21). Antioxidant activities of quercetin and its complexes for medicinal application. Molecules.

[46] Papakyriakopoulou P., Velidakis N., Khattab E., Valsami G., Korakianitis I., Kadoglou N.P. (2022 Aug 18). Potential pharmaceutical applications of quercetin in cardiovascular diseases. Pharmaceuticals.

[47] Elumalai P., Lakshmi S. (2016). Role of quercetin benefits in neurodegeneration. Adv Neurobiol.

[48] Sharma D.R., Sunkaria A., Wani W.Y., Sharma R.K., Verma D., Priyanka K., Bal A., Gill K.D. (2015 Dec). Quercetin protects against aluminium induced oxidative stress and promotes mitochondrial biogenesis via activation of the PGC-1α signaling pathway. Neurotoxicology.

[49] Wang W.W., Han R., He H.J., Li J., Chen S.Y., Gu Y., Xie C. (2021 Apr 20). Administration of quercetin improves mitochondria quality control and protects the neurons in 6-OHDA-lesioned Parkinson's disease models. Aging (Albany NY).

[50] Waseem M., Kaushik P., Dutta S., Chakraborty R., Hassan M.I., Parvez S. (2022 Jan 21). Modulatory role of quercetin in mitochondrial dysfunction in titanium dioxide nanoparticle-induced hepatotoxicity. ACS Omega.

[51] Vargas-Mendoza N., Angeles-Valencia M., Morales-González Á., Madrigal-Santillán E.O., Morales-Martínez M., Madrigal-Bujaidar E., Álvarez-González I., Gutiérrez-Salinas J., Esquivel-Chirino C., Chamorro-Cevallos G., Cristóbal-Luna J.M., Morales-González J.A. (2021 Nov 22). Oxidative stress, mitochondrial function and adaptation to exercise: new perspectives in nutrition. Life.

[52] Wang C.H., Wu S.B., Wu Y.T., Wei Y.H. (2013 May). Oxidative stress response elicited by mitochondrial dysfunction: implication in the pathophysiology of aging. Exp Biol Med (Maywood).

[53] Huang X., Zhao L., Peng R. (2022 Dec 27). Hypoxia-inducible factor 1 and mitochondria: an intimate connection. Biomolecules.

[54] Thomas L.W., Ashcroft M. (2019 May). Exploring the molecular interface between hypoxia-inducible factor signalling and mitochondria. Cell. Mol. Life Sci..

[55] Papandreou I., Cairns R.A., Fontana L., Lim A.L., Denko N.C. (2006 Mar). HIF-1 mediates adaptation to hypoxia by actively downregulating mitochondrial oxygen consumption. Cell Metabol..

[56] Okamoto A., Sumi C., Tanaka H., Kusunoki M., Iwai T., Nishi K., Matsuo Y., Harada H., Takenaga K., Bono H., Hirota K. (2017 Jun 19). HIF-1-mediated suppression of mitochondria electron transport chain function confers resistance to lidocaine-induced cell death. Sci. Rep..

[57] Li H.S., Zhou Y.N., Li L., Li S.F., Long D., Chen X.L., Zhang J.B., Feng L., Li Y.P. (2019 Jul). HIF-1α protects against oxidative stress by directly targeting mitochondria. Redox Biol..

[58] Yu L., Wang Y., Guo Y.H., Wang L., Yang Z., Zhai Z.H., Tang L. (2022 Feb 3). HIF-1α alleviates high-glucose-induced renal tubular cell injury by promoting parkin/PINK1-mediated mitophagy. Front. Med..

[59] Kaneko M., Minematsu T., Yoshida M., Nishijima Y., Noguchi H., Ohta Y., Nakagami G., Mori T., Sanada H. (2015 Sep). Compression-induced HIF-1 enhances thrombosis and PAI-1 expression in mouse skin. Wound Repair Regen..

[60] He R., Wang Z., Cui M., Liu S., Wu W., Chen M., Wu Y., Qu Y., Lin H., Chen S., Wang B., Shao Z. (2021 Nov). HIF1A Alleviates compression-induced apoptosis of nucleus pulposus derived stem cells via upregulating autophagy. Autophagy.

[61] Wang Z., Cui M., Qu Y., He R., Wu W., Lin H., Shao Z. (2020 Oct 15). Hypoxia protects rat bone marrow mesenchymal stem cells against compression-induced apoptosis in the degenerative disc microenvironment through activation of the HIF-1α/YAP signaling pathway. Stem Cell. Dev..

[62] Triantafyllou A., Liakos P., Tsakalof A., Chachami G., Paraskeva E., Molyvdas P.A., Georgatsou E., Simos G., Bonanou S. (2007 Mar). The flavonoid quercetin induces hypoxia-inducible factor-1alpha (HIF-1alpha) and inhibits cell proliferation by depleting intracellular iron. Free Radic. Res..

[63] Radreau P., Rhodes J.D., Mithen R.F., Kroon P.A., Sanderson J. (2009 Dec). Hypoxia-inducible factor-1 (HIF-1) pathway activation by quercetin in human lens epithelial cells. Exp. Eye Res..

[64] Wahyuningsih S.P.A., Dewi F.R.P., Hsan A.S.Y., Lee L.M., Lim V., Aun L.I.L., Ling T.C., Marviella S.T. (2022 Jun). The regulation of hypoxia inducible factor (HIF)1α expression by quercetin: an in silico study. Acta Inf. Med..

[65] Wilson W.J., Poellinger L. (2002 Apr 26). The dietary flavonoid quercetin modulates HIF-1 alpha activity in endothelial cells. Biochem. Biophys. Res. Commun..

